# Public Health Nurses in an Internal Negotiation Process When There Is Concern About the Child’s Care

**DOI:** 10.1177/23333936241267003

**Published:** 2024-08-24

**Authors:** Ingrid Elisabeth Mathisen Haaland, Terese Elisabet Bondas

**Affiliations:** 1 Faculty of Health Sciences, University of Stavanger, Norway

**Keywords:** concern, child neglect, child maltreatment, public health nursing, Norway, bekymring, neglekt, barnemishandling, helsesykepleier, Norge

## Abstract

The aim of the study was to explore and describe how public health nurses at child health clinics experience and perceive the follow-up of children and families when there is concern about the child’s care. The goal was to contribute to knowledge development to guide health-promoting nursing care for children and their families. Theoretical perspectives included health promotion, child-centered and family-centered care, in addition to nursing care. An exploratory qualitative design informed by a hermeneutic approach was used. Data were collected in 3 focus groups with 16 public health nurses and analyzed using latent content analysis. The findings detail public health nurses’ internal negotiation processes in the follow-up of children and the family, and the ways these negotiation processes were influenced by various prerequisites, the approaches for follow-up, dilemmas that affected public health nurses’ approaches, and prolonged dwellings on past responses to children and families of concern. The lack of routines and goals for follow-up, a dominant parental perspective, and ambiguity related to health promotion and disease prevention, all created challenges for the public health nurses. Based on these findings, a model of public health nurse’s follow-up when there is concern about the child’s care was developed for future research.

## Abstrakt

Hensikten med studien var å utforske og beskrive hvordan helsesykepleiere på helsestasjoner opplever og oppfatter oppfølgingen av barn og familier når det er bekymring for barnets omsorg. Formålet var å bidra til kunnskapsutvikling for å veilede forebyggende og helsefremmende sykepleie til barn og deres familier. De teoretiske perspektivene som gav et utgangspunkt og linse for denne studien inkluderte helsefremming, barnesentrert- og familiesentrert omsorg, samt profesjonell sykepleiefaglig omsorg. Et eksplorerende kvalitativt design med en hermeneutisk tilnærming ble brukt. Data ble samlet inn i tre fokusgrupper med 16 helsesykepleiere, og analysert ved hjelp av latent innholdsanalyse. Funnene beskriver helsesykepleieres interne forhandlingsprosesser i oppfølgingen av barn og familien, og hvordan disse forhandlingsprosessene ble påvirket av ulike forutsetninger, tilnærmingsmåter, dilemmaer, og langvarig dveling over tidligere opplevelser med bekymring for omsorgen. Mangel på rutiner og mål i oppfølgingen, et dominerende foreldreperspektiv, og uklarhet knyttet til helsefremming og sykdomsforebygging skapte utfordringer for helsesykepleierne. Basert på disse funnene ble det utviklet en modell til fremtidig forskning, over helsesykepleieres oppfølging når det er bekymring for barnets omsorg.

## Introduction

The focus of this study is public health nurses’ (PHNs) follow-up of children 0 to 5 years and their families in case of suspected or observed deficiencies in the care provided by the parents, but which is not immediately perceived as neglect or so severe that the PHN considers disclosure to the child protective services (CPS). Neglect is conceptualized in this study as a subtype of child maltreatment (CM), in line with the World Health Organization’s definition (WHO, 2022). Neglect is about the child’s basic needs not being met by the caregiver(s), the child’s physical and mental development is therefore at risk (Lundén, 2010).

## Background

Neglect is the type of CM with the highest prevalence, estimated to be 14% to 29% (Clément et al., 2016; Stoltenborgh et al., 2013), with emotional neglect being more widespread than physical neglect (Hafstad & Augusti, 2019; Stoltenborgh et al., 2013). CM is a global public health problem with devastating consequences for children, families, and society (Felitti et al., 1998; Ferrara et al., 2015; Gilbert et al., 2009; Segal et al., 2021; Shonkoff & Garner, 2012; Thoresen et al., 2015). Neglect is regarded as the most damaging form of CM (Clément et al., 2016; Sroufe et al., 2005). The child’s interaction experiences are crucial for brain development, the development of regulation and stress activation (Nordanger & Braarud, 2014), as well as socio-emotional, cognitive, behavioral, and linguistic development (Hildyard & Wolfe, 2002; Naughton et al., 2013; Rocha et al., 2019). The lack of emotional engagement in the child and psychological unavailability are highlighted as the most harmful forms of neglect (Egeland, 2009; Sroufe et al., 2005), while at the same time they are most easily overlooked (Killén, 2019).

The UNICEF (1989), states that children have the right to protection and care that ensures the child's well-being (Art. 3), the right to protection against all forms of maltreatment (Art. 19), and the right to development, health, and health care (Art. 6, 24, and 27). The best interests of the child shall be the fundamental consideration in all matters (Art. 3).

### Previous Research

PHNs find it a difficult task to help children who may be victims of CM, as they doubt their observations and assessments, and feel inadequacy and fear upon acting on suspicion (Albaek et al., 2018; Dahlbo et al., 2017; Midtsund et al., 2023; Söderman & Jackson, 2011; Visscher & van Stel, 2017). Especially less obvious forms of CM are challenging for professionals to detect (McTavish et al., 2017). Addressing one’s concerns with the parents is perceived as challenging for PHNs (Dahlbo et al., 2017; Engström et al., 2021; Midtsund et al., 2023; Poutiainen et al., 2015). PHNs experience inadequate resourcing as a barrier in their work with CM (Barrett et al., 2024; Kent et al., 2011; Midtsund et al., 2023). Visscher and van Stel (2017) found that the PHNs’ follow-up of CM is significantly suboptimal, with a large variation in individual practices. Engström et al. (2021) showed that there is a significant gap between PHNs’ perception of the importance of helping families with psychosocial difficulties, and their actual help and guidance. PHNs themselves have described tailoring their follow-up to the individual family’s need and offering additional appointments when parents experience difficulties (Barrett et al., 2024; Midtsund et al., 2023). However, PHNs often experience a lack of knowledge, and may feel inadequate in assessing and promoting a good parent-child interaction (Eklund et al., 2022). A good relationship with parents is essential to support the family and help the child in PHNs’ view (Barrett et al., 2024; Browne et al., 2010; Dahlbo et al., 2017; Einboden et al., 2019; Eklund et al., 2022; Kageyama & Yokoyama, 2018; Midtsund et al., 2023; Paavilainen & Åstedt-Kurki, 2003; Söderman & Jackson, 2011), but can also act as a barrier to detecting and following up on CM (Schols et al., 2013).

Lines, Kakyo, Grant, and Hutton (2023a) found that nurses’ and midwives’ contributions to a public health response to CM are almost invisible in policies concerning child protection, health, and development. In line with this, Einboden et al.’s (2019) study shows how nursing contributions to CM prevention is overlooked, and they highlight the potential for a more significant role for nurses in a public health approach to child protection. There is a lack of research on PHNs’ experiences and perceptions regarding the follow-up of children and families when there is concern about the child’s care. Previous studies have focused mainly on prevention, detection, and reporting of CM.

### Theoretical Perspectives

Several theoretical perspectives provided a starting point and lens for this study and included perspectives related to health promotion, child-centered and family-centered care, in addition to nursing care. In this study, nursing was viewed from a relational perspective that is based on caring and ethics (Delmar, 2021). Responsibility for the weak was a guiding value principle, based on the nursing philosophy of Martinsen (2003). Delmar (2021), extending Martinsen’s and Løgstrup’s philosophy of care, views caring and responsibility as two sides of the same coin in nursing. Both caring and responsibility contain an imperative for action, in which the nurse must perceive and understand the appeal for help.

We conceptualize PHN as prioritizing family- and child-centered care. Family-centered care assumes that the family and the child’s needs must be seen as a whole, and that the child’s well-being is best served when the PHN supports the parents’ ability to meet the child’s needs (Coyne et al., 2018). In child-centered care, the child is at the center of thinking and practice, with the child’s own rights, competence, dignity, and voice all being strongly emphasized (Coyne et al., 2018).

The primary objective of public health nursing is to promote health and to prevent disease (Dahl, 2020) in a relational and salutogenic way (Wightman et al., 2022). Garner and Yogman’s (2021) ecobiodevelopmental model for illness and well-being provides a theoretical background to understand the health promoting and disease-preventive role of PHNs in the follow-up of children living in inadequate care. The model highlights that safe, stable, and nurturing relationships are biologically necessary for all children, as these relationships reduce the toxic stress responses that follow adverse events and upbringing, while at the same time building future resilience (Garner & Yogman, 2021).

These theoretical perspectives guided the study including data collection. We returned to these perspectives to deepen the understanding in the discussion, acknowledging the openness that is required in a qualitative exploratory study (cf. Bradbury-Jones et al., 2014).

## Aim

The aim of the study was to explore and describe how public health nurses at child health clinics experience and perceive the follow-up of children and families when there is concern about the child’s care. The goal was to contribute to knowledge development to guide preventive and health-promoting nursing care for children and their families.

## Method

### Design

We chose an explorative qualitative research design (Elo & Kyngäs, 2008; Elo et al. 2014), that involved a human science epistemological basis of interpretation using the notions of a hermeneutic circle and pre-understanding to create understanding, inspired by Gadamer (1989). A hermeneutic approach is not restricted to specific methods but embodies an understanding of the participants in a research study as meaning giving in context. Interpretation is necessary to understand meaning. According to Gadamer (1989) there is no objective value-free position and the researchers’ values are therefore reflected and recognized during the whole research process. We approached this research as an experienced qualitative researcher (T.B.) and a novice (I.E.H), which created a questioning attitude and fruitful dialogues. We have reflected on our varied knowledge, and personal and professional public health nursing experiences both as professionals and mothers to help clarify our pre-understanding during the research process and in research seminars, as the prior understanding can affect all stages of the research process (Malterud, 2017).

### Context

The Norwegian child health clinic (CHC) program is free and voluntary and consists of 14 stipulated consultations for all children from 0 to 5 years. In 2022, 98% of all 8-week-old and 4-year-old children were examined at the CHCs (Statistics Norway, 2023). PHNs have a mandatory duty to report to the CPS when there is reason to believe that a child has been subjected to severe neglect (Health Personnel Act, 1999, § 33). When PHNs encounter less severe neglect that is not covered by mandatory reporting, they themselves must ensure that a follow-up is offered to the family (Regulations on Child Health Clinics and School Health Services, 2018, § 5d). However, the guidelines do not describe in any detail how this follow-up should be conducted (Directorate of Health, 2024).

### Recruitment, Participants, and Data Collection

Guided by a purposive sampling strategy based on Elo et al. (2014), a total of sixteen female PHNs agreed to participate in the study. Public health nursing is a female dominated specialty and in Norway, there are only a few male PHNs. In the planning phase, the recruitment process, and data collection, we were three research teams that collaborated. To recruit participants, the researchers called the managers at eight different CHCs in three different sized municipalities in one Western Norwegian County. The managers were asked to forward the study invitation to their staff via email. The interested PHNs then replied to the first author directly by phone or email, or through their manager.

The inclusion criterion was a qualified PHN within the ordinary CHC program. We conducted three FGs (FGs) with six, four, and six participants, respectively (Table 1).

**Table 1.** Overview of Participants.

Focus group	Participants	3–5 years’ experience	6–9 years’ experience	10–19 years’ experience	20–28 years’ experience
1	6	1	2	1	2
2	4	—	—	4	—
3	6	3	—	1	2
Total	16	4	2	6	4

**Figure 1.**
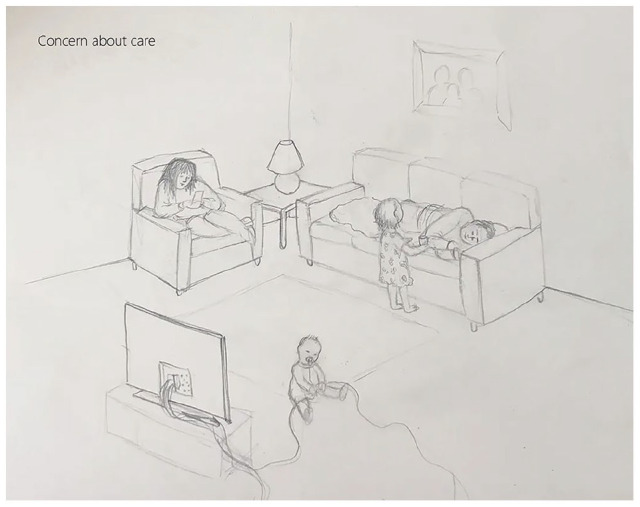
Vignette (by I. Mathisen Haaland).

The focus group discussion guide included presentation of a vignette (Figure 1) to support interaction among participants (Liamputtong, 2011) and to capture various public health nursing practices, including both individual and shared understandings on this complex topic.

A pilot FG with two PHNs was conducted to try out the vignette and the interview questions. The FGs were conducted over the course of 3 weeks in October 2022, and took place at three different CHCs. Each FG lasted for approximately 90 min, of which the part represented in this study lasting for approximately 30 min. As an entrance to the FG, the first author presented a vignette to introduce the informants to the topic (see Figure 1).

According to Hughes and Huby (2001), a vignette can function as a stimulus for group discussions and is particularly useful for difficult and complex topics. The use of vignettes can also improve the quality of data by creating a distancing effect, thereby reducing the risk of informants’ socially accepted responses (Hughes & Huby, 2001). The vignette was developed by the first author, noting down key words about child neglect and PHNs’ encounters with CM from previous research and literature. These key words (deficiency state, inaccessibility, physical/emotional neglect, diffuse) were the starting point for an ambiguous drawing of a family situation. Our vignette was validated through pilot testing with PHNs, as well as assessed with consideration for clarity, relevance, and importance (St. Marie et al., 2021). The starting point for the interview questions was an interview guide with room for flexibility (Supplemental File 1—The Interview Guide). The first author conducted the FGs. Audio recordings were subsequently transcribed by the first author.

### Analysis

**Figure 2.**
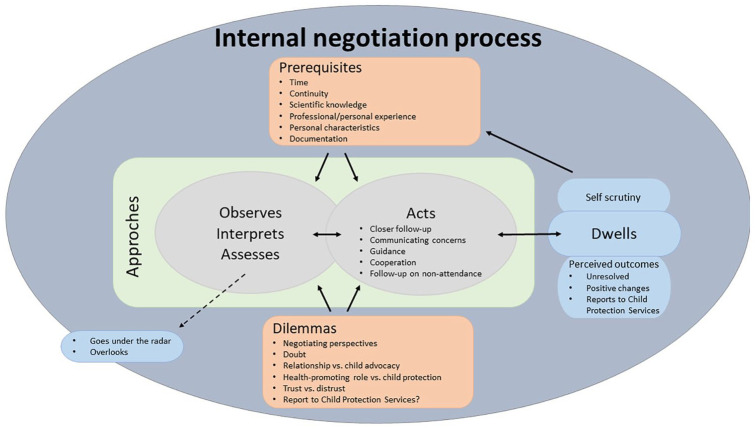
The internal negotiation process when there is concern about the child’s care.

A qualitative latent content analysis according to Elo and Kyngäs (2008) was chosen because of its potential for an explorative study on a topic with scarce and fragmented knowledge, and for the potential of creating conceptual models based on empirical data. The inductive analysis process consisted of three phases: (a) The preparation phase: Becoming familiar with the text during transcription and repeated reading; (b) The organization phase: Started with open coding, in which thoughts and ideas were repeatedly noted in the text during reading and analysis. The first author was responsible for the analysis and the second author followed the categorization with discussions and subtle changes during the analysis. Categories were generated and re-grouped several times to create main, generic, and subcategories (Supplemental File 2—Overview of the Categories), with each category being named, and illuminated with a citation. The main category tied the categories together. The analysis can be described as a hermeneutic circle of description and interpretation between parts and a whole for understanding. During this analysis process, the contours of a model (Figure 2) were created and refined in the back-and-forth process of reporting in phase 3. The findings were described, with a thorough reflection on citations to help illuminate each category and the preliminary model (Figure 2) has been visualized for further research.

### Research Ethics

We have rigorously followed the Declaration of Helsinki (World Medical Association, 2013) and relevant legislation (Health Research Act, 2008; Research Ethics Act, 2017; Personal Data Act, 2018). Participation was voluntary and based on informed consent. The participants were informed in writing about the study, their right to withdraw from the study without consequences at any time and contact information to the responsible researchers. The Norwegian ethics committee has approved the study, SIKT (163443). Confidentiality, by ensuring the de-identification of personal data and anonymization in the presentation, was ensured.

## Findings

In the analysis, the following four generic categories and their subcategories were constructed to describe the PHNs’ experiences and perceptions of follow-up of the child and the family: (a) *Prerequisites* for the PHNs’ follow-up on care concerns, (b) The PHNs’ *approaches* to follow-up when concerned about care, (c) *Dilemmas* that affect the PHNs’ approaches, and (d) The ongoing *dwellings* of PHNs during and after follow-up. The main category that was interpreted from the generic categories and their subcategories was named, “*An internal negotiation process*.”

### An Internal Negotiation Process

The findings showed that the PHNs’ follow-up of the child and the parents was not a linear process when they were concerned about the child’s care at the CHC, and it did not follow any routine. The follow-up was characterized by an ongoing internal negotiation process that included different prerequisites, approaches, dilemmas, and PHNs way of dwelling on previous experiences. A model was developed based on the findings (see Figure 2). In general, the follow-up process started with varying *approaches* for follow-up that were based on worrying observations. From the PHNs’ observations, interpretations, and assessments the process could either stop or progress into acting. The process could further develop toward an outcome or go backwards again, and often both ways simultaneously. Findings showed that there were fluid transitions between different approaches, and it appeared random and person dependent as to which actions were taken. The follow-up process was continuously influenced by *prerequisites.* Inherent *dilemmas* related to the follow-up of neglect, ethical dilemmas and dilemmas related to the role of PHNs and their professional mandate, characterized the process.

The invisible internal negotiation process—especially the negotiation around perspectives and concern/not concern—seemed to be a steering background process that influenced the follow-up. Findings showed that this internal negotiation process also continued long after the meetings with the family were over, even when the PHN had finished following the family as *dwellings*. In the model, it is stated that self-scrutinizing was an “end product,” which further became a prerequisite; PHNs experienced learning from their mistakes despite uncertain outcomes.

### Prerequisites

From the PHNs perspectives, *prerequisites* had a continuous impact on the follow-up when they had concern about a child’s care. This generic category was based on the following subcategories: Time, continuity, scientific knowledge, professional and personal experience, personal characteristics, and documentation.

#### Time: “The Timeframes Are Crucial”

The PHNs experienced that the timeframes were decisive for the quality of the follow-up in cases of concern about care. Time pressure affected the PHNs’ experienced capability to perceive and capture concerns in the consultations. Additionally, the PHNs described having to let worries go when it was busy because they did not have time to follow-up:

When it is busy, it is much easier to feel like, “What was it that I saw?” You just think, “No. . .,” you just let it go in a way. (FG1)

Being in an unresolved follow-up process was described as in limbo, evoking strong emotional discomfort. During busy workdays, the PHNs found it difficult to prioritize time to pause and reflect on what their observations and own reactions were about. However, this reflection time was crucial to be able to understand the concern, and in turn, such clarification made action possible according to the PHNs.

#### Continuity: “And Then Came Summer”

Having the same PHN working with a child from birth up to 5 years of age and his/her family was highlighted as an important prerequisite for follow-up. The relationship with the family was experienced as stronger if there was this continuity of care. The PHNs felt better able to notice signals, notice changes, and dare to directly address concerns based on a more in-depth understanding what the family was struggling with. One of the PHNs described the difficulties she experienced grasping the family’s challenges without continuity of care:

They just come to you, and then you are left with: “Who was this?”, and you are sitting there like: “What was this?” (FG1)

In cases where several different PHNs had been involved, because of holidays, sick leave, or high turnover, the PHNs described that the follow-up suffered. This affected vulnerable families and children, who were at risk of slipping under the radar.

#### Scientific Knowledge: “Knowledge Has Made Me Tougher”

The PHNs emphasized the importance of having scientific knowledge about what neglect is, about prevalence and about risk factors to effectively identify and follow-up on child neglect. They described having changed their practice based on new knowledge acquired through the government initiatives on CM, and they experienced that increased knowledge made them tougher in handling concerns about care, because the knowledge provided them with a kind of support and protection. The usefulness of different theories and models in supporting the PHNs follow-up with children and families was highlighted, but the PHNs stated that they had to “own” the theory or model for it to be useful in practice. It was evident from some of the PHNs’ statements that theories could be misinterpreted in a way that did not appear to protect children, for example, by referring to children’s ability to adapt to the care they receive.

#### Professional and Personal Experience: “In Our Backpacks”

Experienced PHNs described the difficulty they faced following up on concerns about care when they were newly graduated, whereas the less experienced PHNs described how they felt to be insecure. The PHNs pointed out the importance of being aware of their own vulnerability, and tried to understand the impact of their own history and professional view on their follow-up:

We have our own experiences in our backpacks. And practice experience and interpretation of the profession. And we wouldn’t necessarily say that it was neglect on the same things. (FG3)

The fact that the follow-up was person-dependent was also implicitly evident from the practice examples.

#### Personal Characteristics: “We Must Be Courageous”

The PHNs’ examples indicated that the follow-up and protection of vulnerable children depended to some extent on whether the child and the family happened to be identified and/or followed up by courageous and skilled individuals. These were well-prepared, attentive, knowledgeable, and fearless. The practice narratives of the PHNs clearly demonstrated how courage was a necessary quality to be able to follow-up, and they also showed how a lack of courage affected vulnerable children who then did not receive follow-up.

#### Documentation: “It Matters a Lot What Is Written in That Medical Record”

Proper documentation was highlighted as important to ensure that any concern did not go under the radar, and important in case the PHNs later had to report to the CPS. The interaction was the most critical issue to document. This indicates that the PHNs perceived the interaction between the child and the parent to be the most important indicator of the child’s care situation. The PHNs said that they compared their observations and assessments to what had been previously journaled in the medical record, and these earlier records had a significant impact on further follow-up. In particular, the less experienced PHNs began to doubt themselves, if they did not find support for their own observations in the records. Documentation was also described as a way for the PHNs to “*work through*” their observations and gut feeling.

### Approaches

The second generic category captures the PHNs’ approaches when they are concerned about care, beginning with observing, interpreting, and assessing. From there the PHNs could start to act in various ways.

#### Observing, Interpreting, and Assessing: “So I Felt Uneasy, I Didn’t Want to Just Let It Go”

The PHNs told that when they were concerned, they observed the child, the parents, and the interaction. Especially signs and behavior in the child, which could indicate attachment difficulties awakened or enhanced the PHNs’ concern and led to follow-up. In the observations of interaction, the PHN was particularly concerned if the parents were unavailable, that is, did not pay attention to the child and did not respond when the child sought them. PHNs’ concern was also raised when parents did not attune sensitively to their child and if parents were unable to comfort their child. The PHN described the worry that arose as an uneasiness, as a gut feeling. This uneasiness led the PHN to not let go, but instead to follow up the child’s care.

Some of the PHNs described assessing alternative approaches and would also try creative measures to “*get the parents on board*” without involving CPS, called “*the classic one*.” Other PHNs were unsure how they could best help the family, and sometimes they felt helpless and seemed to get stuck in the observation phase. Several pointed out that the assessment of the observations and the assessment of possible further follow-up were based on individual judgment.

#### Acting

The PHNs described taking more active measures to follow up when the concern for care increased, including closer follow-up, communicating concerns with the parents, guidance, cooperation, and follow-up on non-attendance. Sometimes the PHNs stopped this acting after a while because the concern decreased, they then went back to observing and assessing.

#### Closer Follow-Up: “Simply Follow Them More Closely”

When concerns about care arose, the PHNs stated that they provided follow-up through more frequent contact. They scheduled extra consultations and/or called the parents. The extra consultations aimed at creating a relationship with the parents to get into a better position to map their concern further and to monitor the child’s development. Inviting parents to extra consultations was described as easy in cases where parents themselves experienced challenges. When parents wanted distance, the PHNs described having to “*work to bring them back*,” and often they failed. The PHNs emphasized the importance of being persistent to have the family come back in the child’s first year of life since the CHC is the only certain point of contact outside the family before the child starts day care.

#### Communicating Concerns With the Parents: “Direct With Love”

Words like “*tough*” and “*to dare*” were used when PHNs talked about addressing concerns—indicating that they experienced it to be unpleasant or scary. The PHNs communicated their concerns either directly or indirectly. In contrast with indirect addressing, the PHN gave parents the opportunity to participate, and provide their own views on the matter by being direct. The PHNs pointed out that a direct approach ensured that the parents did not have to guess what the PHN was looking for or expecting. Being clear, direct, concrete, and tough was highlighted as a kind of “*gold standard*” or a goal in the development as a PHN. At the same time, the PHNs described the importance of presenting their observations wisely to the parents, so as not to lose the relationship and the possibility to reach the family with follow-up:

We can be direct in many ways. We can be direct with love. (FG3)

#### Guidance: “To Nurture the Attachment”

The PHNs described guiding the parents in diverse ways during consultations to enable the parents to meet their child’s care needs in a better way, and to improve the parent-child interaction: “*sow some seeds.*” Always articulating the child’s observed attachment behavior specifically to the parents was highlighted as important in making the parents sensitive to their child’s needs. Through the “*power of example*,” the PHNs tried to be a role model for the parents by engaging with the child in an attuned and responsive manner, with the PHNs describing spending time close to the child, or even on the floor with the child. PHNs also provided specific advice to parents or instructed parents directly during the consultation. All the PHNs agreed that emphasizing existing resources and praising what worked was an important starting point in the guidance:

To nurture, to nurture the attachment is something we often say. We must think, that even if it’s a bit weak, we should nurture what we see in mother, so that she does more of it. (FG3)

#### Cooperation: “Multiple Agencies Can Fail”

When concern about care arose, the PHNs often contacted the kindergarten to gather more information. The PHNs sometimes called CPS and discussed the case anonymously seeking advice. Municipal mental health services and municipal family counseling were also highlighted as collaborative partners that the PHN could refer to. The follow-up of the family often seemed to fail here, if the family did not show up or soon stopped seeing the municipal helper, while the PHN was not always informed that the family was without follow-up. In some cases, the PHN was not taking responsibility if other agencies had been involved, believing that “*someone else*” should provide better follow-up. The PHNs described how several agencies somehow failed to follow up on neglect, or that poor routines and lack of collaboration had led to an insufficient follow-up of children.

#### Follow-Up on Non-Attendance: “Have Been Out Searching”

The PHNs knew that the CHC should have an established procedure if a family did not attend or canceled appointments repeatedly, but they were uncertain about the implementation. The PHNs said that they called the parents if they did not show. If the family did not attend the scheduled consultation, several of the PHNs told that they went to the family’s home without giving prior notice. This was highlighted as both an important and appropriate way to follow-up to find out where the family was and how they were doing. At the same time, the PHNs perceived this practice as illegal.

### Dilemmas

The third generic category illuminates the dilemmas that affect PHNs’ follow-up of concern about the child’s care based on the following subcategories: Negotiating perspectives, doubt, building relationships versus child advocacy, health-promoting role versus protecting children, trust versus distrust, and reporting to CPS.

#### Negotiating Perspectives: “Team With the Mother”

The PHNs seemed to negotiate three different perspectives when concerned about care: The parental perspective, the child perspective, and the public health nursing perspective. The negotiation was primarily an internal process but could also involve colleagues.

The parental perspective—that is, the empathy and understanding of the parents’ difficulties, especially the mothers’ struggles—was clearly the most dominant among the PHNs. This was evident in the PHNs’ creation of excuses that could explain why the parents did not adequately attend to the child’s needs. There was normalization and trivialization of parental behavior described as harmful in the professional literature. In some cases, the PHNs lost sight of the child’s situation because of the strong empathy with the mother:

During the 3-month check-up, the mom told me that she had deceived me on that EPDS assessment. That over the summer, she had thoughts of giving the child up for adoption and that she felt unable to go on. The chaos she experienced was like a life crisis. The child’s father had promised to support her, urged her not to have an abortion [. . .] but then he backed out. [. . .]. I felt like I was really on her team, you know? I spoke with the crisis center, discussed how she could get legal help, how she could contact the family counselling office, and how she could see a general practitioner. (FG1)

There was no indication in this practice narrative (where this excerpt comes from) that the PHN assessed the child’s individual needs for follow-up while living with a single caregiver in an ongoing crisis.

The child perspective was reflected in how the PHNs tried to see the world from the child’s point of view and tried to understand the child’s behavior as communication. Some of the PHNs stressed that the child’s needs should be prioritized, even when they clashed with their parents’ needs.

The public health nursing perspective was reflected in the way in which the PHNs negotiated their observations in relation to their professional and experiential knowledge. This perspective was also evident in the role-related negotiations regarding PHNs’ areas of responsibility, and in the ethical reflections on trust, responsibility, and relationships.

#### Doubt: “It’s Kind of Vague”

When the PHNs discussed the vignette and shared situations from practice, it was clear that there was also a negotiation consisting of arguments that either supported or diminished their concern. The fact that the same family could exhibit both worrisome and “*normal*” signs seemed to confuse the PHNs. It was common for PHNs not to trust their own observations when the family exhibited what the PHNs perceived as vague or “*mixed signals*.”

Although the PHNs pointed out that there was a “*certain professional standard*,” determining what constituted “*good enough*” care was experienced as a difficult assessment, leading to self-doubt. The PHNs were also careful to acknowledge that what they observed of the child and family at the CHC could be influenced by the context. Several PHNs seemed to doubt whether the position—with the consultation model at the CHC—was suitable for identifying and following up on children living in failing care:

We’re keeping an eye on things, you know. We have all these snapshots. Things can be situation-dependent, after all. (FG2)

#### Building Relationships Versus Child Advocacy: “Does the Child Have Time to Wait?”

An obvious dilemma when following up on concerns was the balancing act between building relationships with the parents and being the child’s advocate. The PHNs considered trust and a good relationship with parents to be the key factors in being able to reach and support the family, and that the alliance with the parents helped the child indirectly in the best way available.

However, a couple of PHNs pointed out that the slow relationship-building strategy was not always the best for the child, and that the consideration for the child’s development should take precedence:

It’s a bit of a difficult balancing act with building relationships. Because that’s about reassuring the parents. But at the same time, if it is a young child who has begun to turn away, then the child does not have much time. So, we always must think, does the child have time to wait for this? (FG2)

#### Health-Promoting Role Versus Protecting Children: “The CHC, It’s a Safe Space, a Positive Word”

Having a health-promoting role with a focus on resources and coping skills, while at the same time having a disease preventive role emphasizing risk factors and deviation, proved to be another dilemma in the PHNs’ follow-up. It emerged through the PHNs’ practice examples that a one-sided health-promoting approach could lead to trivialization and speculation about mitigating circumstances. The need to convey positivity to parents also seemed to blind the PHNs from clearly seeing the signals pointing toward inadequate care. The PHNs themselves were proud of the health-promoting role they and the CHC have in society. The PHNs communicated both implicitly and explicitly that focusing on the positive was an indisputable part of the PHNs’ role, and therefore differed from the CPS’ approach:

Trying to end with something positive when they walk out the door, I think most of us do that, no matter how bad things may seem. Lift them up a little as they leave the office, in a way. (FG2)

Striving to safeguard the societal positive view of the CHC and its voluntary services also proved to create a conflict with the PHNs’ duty to protect and advocate for the child.

#### Trust Versus Distrust: “We Are Deceived, Most Certainly”

Another dilemma that emerged was that the PHNs had to have trust in the parents as a starting point, even if they had experienced that parents could withhold information or answer falsely. Some PHNs highlighted examples, in which parents actively had hidden problems in the family or problems with their child’s development. However, these stories revealed that the PHNs often perceived worrying signals from the child and parents and had a gut feeling that something was not right. Despite this, follow-up measures had not been taken, as the parents had stated that everything was fine.

#### Reporting to CPS: “I Don’t Find It That Unpleasant, If That Reporting Duty Is Unleashed”

An ongoing and difficult assessment in the PHNs’ follow-up was to determine how long they themselves should follow up, and how to know when the duty to report had been unleashed. The PHNs described that they could feel relief when the reporting obligation to the CPS had been triggered, because they as PHNs then could hide behind this duty. The PHNs felt unprotected, burdened, and alone with their responsibilities, when they were following up on their own.

### Dwellings

The fourth generic category describes the PHNs prolonged dwellings about past follow-ups, based on the following subcategories: Scrutinizing myself and perceived outcomes.

#### Scrutinizing Myself: “I Will Never Forget”

In the FGs, the PHNs appeared open and scrutinizing, critically reflecting on situations where they only later had realized the severeness of the child’s care situation. The PHNs said they had learned from cases where they had not noticed or understood the signals from the child or parents, where they had made incorrect assessments, or had not followed up well enough. It also emerged that these cases weighed heavily on the PHNs and got stuck in their minds. Several of the PHNs reflected on how they had changed their practices after making mistakes, and made suggestions as to what they should have done differently or would do in the future if they encountered something similar:

When I think back on it, I think that that mother, that body language, I’m going to have to do a lot more [in the future] to have her participate in parental guidance. I'm not going to give up! (FG3)

In hindsight it was clear to several of the PHNs that they should have conducted home visits to some of the families of concern, as it would have provided greater insight into the actual situation of care.

#### Perceived Outcomes: “Haven’t Quite Landed This Yet”

Following up the child’s care was described by PHNs as a “*long process*,” and “*difficult to reach the goal*.” The PHN could observe signs of severe neglect over a lengthy period—passive parents, increasing developmental disparities in the child, parents attempting to hide problems and not wanting further help—but it could still be difficult for the PHN to “*land*” on the right course of action. “*To hurry up slowly*” and “*play it cool*” were highlighted as desirable attitudes from the PHNs’ perspective.

Sometimes, the PHNs experienced that the follow-up had been fruitful, or led to positive changes. However, most of the practice examples were about a follow-up that had not led to improvement. The child and family’s situation remained unresolved, or that after a period of follow-up, the PHN had to report concerns to the CPS. In both cases, the PHN seemed to continue to dwell on the concern. The PHNs continued with different approaches, alongside the dwellings, if they decided not to report to the CPS.

## Discussion

Findings in this study show that the PHNs undergo an internal negotiation process, not previously described, to clarify their vague concerns about care that may arise in the encounter with children and parents at the CHC. Our findings show that the ongoing process of internal negotiation in the follow-up were influenced by prerequisites for follow-up on child care concerns, the approaches for follow-up, dilemmas that affected PHNs, and PHNs’ prolonged dwellings on previous responses to children and families of concern. With no established routines around the concerns for care, variation in PHN practice will inevitably leave some children with a less secure safety net than other children. In the following sections, we will use the theoretical perspectives of nursing care, family centered and child centered care, and health promotion and disease prevention to deepen understanding of PHN practice in this context.

### Public Health Nursing When There Are Concerns About the Care

One way of understanding the PHNs’ inner negotiations in the follow-up is by means of Martinsen’s (2014) description of the interaction between sensing and understanding. Sensing provides an independent gateway to the world, regardless of understanding, while understanding creates a distance and overview, and is linked to our prior understanding (Martinsen, 2014). In the inner negotiation process, which this study reveals, it appears that the PHNs’ concern for the child increases when they actively connect knowledge about neglect to their sensations and specific observations. Professional knowledge appears to help the PHNs to understand what they are sensing, and with a greater understanding the PHNs describe that their courage and ability to act also strengthen.

Nonetheless, it appears that the PHNs in this study doubt what sensing can provide them in terms of “truth”; they find it difficult to trust their gut feeling, and to assess concerns based on observations and interactions with the child and family during consultations. Yet, sensing is a necessary tool in nursing (Martinsen, 2014) and, in this case, to understand what the child and family are struggling with if parents do not disclose. Both Killén (2019) and Lundén (2010) emphasize that open, attentive, and knowledgeable professionals can detect child neglect by paying attention to the signals that the child and parents inevitably send out. Erisman et al. (2020) concludes that intuition is an integral part of the work of CHC-practitioners, and by using intuition nurses can act upon signs earlier, which is of great importance to prevent CM and start early interventions.

Our findings parallel Kent et al. (2011) and Midtsund et al. (2023), showing that prerequisites, like appropriate resources for public health nursing are crucial influencers on PHNs likelihood of following up their concerns, and how this follow-up is done. Neumann (2009) finds that PHNs have a tradition of accepting the burden of responsibility for significant societal issues that they cannot solve alone and argues that this tradition has its roots in gendered ways of working. Albaek et al.’s (2018) study on the other hand finds that PHNs and other professionals working with CM seem to externalize the emotional difficulties they experience, by requesting more resources, guidance, knowledge, guidelines, and assessment tools—rather than asking for interventions to help them regulate emotional discomfort or strategies for handling dilemmas and uncertainty.

The importance of continuity to identifying at risk children and follow-up care is not described in previous research. The findings indicate a vulnerable CHC service for the children and families of concern in the study setting, because of the lack of clear routines for ensuring follow-up. Follow-up in the case of concern about the child’s care seems to be person-dependent, which is in line with previous research on CM (Visscher & van Stel, 2017). In this study, the variation in follow-up seems to be influenced by the differences in the “backpacks” of individual PHNs, that is, the differences in professional knowledge, personal qualities, and professional and personal experience. This is problematic, as all children have an equal right to health and development through the UNICEF (1989). The variation in practice will also be an obstacle to reducing social inequalities in health, which is a stated purpose of CHCs in Norway (Regulations on Child Health Clinics and School Health Services, 2018, § 1d). However, the PHNs in this study appear more reflective about the practice variation than previous research has described (Kent et al., 2011; Lines et al., 2019; Schols et al., 2013).

Like previous studies (Dahlbo et al., 2017; Engström et al., 2021; Midtsund et al., 2023; Poutiainen et al., 2015; Schols et al. 2013), the PHNs in this study found it difficult to raise concerns with parents. Visscher and van Stel (2017) argue that improving the communication should be a top priority and is a crucial element in preventing CM. Findings in this study show that the PHNs experienced the direct communication as having benefits for all the parties involved. At the same time the PHNs wished to present their observations to the parents in a “wise manner” so as not to lose the family. Parallelling the PHNs in Barrett et al.’s (2024) and Midtsund et al.’s (2023) studies who found it demanding to balance parent support and safeguarding of the child, our findings show that it is a challenging balancing act for PHNs to take care of the parents’ vulnerability and at the same time be direct enough to bring about a constructive dialogue. However, our findings point toward an experience-based insight among PHNs, that being respectfully direct, and caring need not be contradictory.

### Family-Centered and Child-Centered Care

The PHNs’ follow-up in cases of concern about the child’s care most often resembled Coyne et al.’s (2018) description of family-centered care. Coyne et al. (2018) highlight the importance of health personnel being able to understand the strengths and weaknesses of family-centered and child-centered care and having the skills to choose the right approach in individual situations. Based on the findings, the PHNs did not appear to make conscious choices regarding perspective. Family-centered care is a parent-focused approach and assumes that the needs of the family and the child are the same (Coyne et al., 2018). This study finds that a one-sided family-centered approach can cause the PHN to lose sight of the child’s best interests. The findings show how deeply some PHNs immerse themselves in and want to alleviate the mothers’ situation, and in doing so, they can forget the potentially serious consequences for the child. Neumann (2009) found that PHNs with references to their own motherhood express a deep understanding of mothers’ suppressed position and, as a result, a mother’s limited ability to care for her child.

A clear stance for child-centered care is reflected in the statements of a minority of PHNs in this study, who emphasize that the child’s needs must be supreme. By asking: “Does the child have time to wait for this?,” the PHN prioritizes the best interests of the child in accordance with the UNICEF (1989). In addition, through this question, the PHN assumes responsibility for the weak, an overarching value principle in nursing (cf. Martinsen, 2003)

The findings show that PHNs are doubly challenged when the child and the parents are signaling conflicting needs, a finding paralleled by Skarsaune and Bondas (2016) and Lines, Kakyo, Hutton, and Grant (2023b). The parents can convey a desire for autonomy and distance from the PHN, for example by declining extra consultations, while the child’s behavior may signal an appeal for the PHN to closely follow the development. The appeal implies a responsibility and a requirement to act (Delmar, 2021). For PHNs to act on conflicting appeals, it is necessary to first determine whose appeal is more significant. Based on the theory of parallel processes (Killén, 2019), it can be argued that family-centered care should be the primary perspective. In the findings, family-centered care nevertheless gave way in favor of child-centered care when the appeal from the child became too strong. Findings in the study indicate that this shift in perspective often occurs too late, or not until self-examination processes follow an unsuccessful follow-up. The responsibility and demand for action related to the child’s appeal—the weak—have then not been adequately taken care of by the PHN, and the child’s developmental opportunities may have been curtailed (cf. Delmar, 2021).

### Health Promotion and Disease Prevention

The study finds that health promotion emerges as the core approach, through the emphasis on relationship-building and a focus on the positive, resources, and coping skills. Health promotion and disease prevention are often highlighted as complementary approaches in the public health field (Mæland, 2016). In this study, however, these two strategies sometimes proved to be in conflict, and created dilemmas in the PHNs’ follow-up. The strong emphasis on the positive seemed to prevent PHNs from perceiving obvious risk factors and neglect. This is in line with Neumann’s (2009) findings, which indicate that the PHNs’ normalization mandate hinders the detection and response to deviations. Another way of interpreting the PHNs’ constant pursuit of the positive and ignoring of the signs of inadequate care can be about survival strategies that professionals use to protect themselves against unpleasant feelings (Albaek et al., 2018; Killén, 2019).

Our study shows that the PHNs’ health-promoting approach can be summarized as relationship promotion. This includes strengthening the interaction between parent and child, as well as building relationships between the PHN and parents. The PHNs’ emphasis on relationship is in line with Garner and Yogman’s (2021) ecobiodevelopment model, which conveys how crucial it is for children to live in safe, stable, and nurturing relationships. Based on Garner and Yogman’s (2021) theory, PHNs can also strengthen children’s relational health by creating safe, stable, and nurturing relationships with parents, in line as well with Killén’s (2019) parallel processes.

In health promotion, the process is just as important as the outcome (Mæland, 2016). Still, in matters relating to children's early development, it is controversial to equate the process with the outcome, given our knowledge of the grave consequences of neglect. One problem that the findings reveal is that the slow relationship-building process with the parents may conflict with protecting the child and may jeopardize the child’s development. In all previous research on PHNs’ follow-up of CM, the importance of relationship building with parents has been highlighted (Barrett et al., 2024; Browne et al., 2010; Dahlbo et al., 2017; Einboden et al., 2019; Eklund et al., 2022; Kageyama & Yokoyama, 2018; Midtsund et al., 2023; Paavilainen & Åstedt-Kurki, 2003; Söderman & Jackson, 2011). Nevertheless, Schols et al. (2013) find that the relationship with parents can also function as a barrier in helping the child. In our study, some PHNs describe how views on relationship building can change at a certain point in the follow-up process. We found that health promotion—in the form of relationship building—seems to be the most important strategy until the concern reaches a certain point, at which the disease prevention perspective becomes more important. Health promotion versus disease prevention and family-centered care versus child-centered care thus appear to follow similar paths in the follow-up process. Health promotion—like family-centered care—seems to be a more fundamental and a long-term strategy in the work of PHNs, while disease prevention functions as a kind of crisis plan, such as child-centered care.

Neglect is not clearly mentioned in the numerous plans of the Norwegian authorities dealing with combating CM. It is worrying that the most prevalent form of CM, which also has the greatest negative impact on children’s development, is not highlighted to a greater extent. Ramiro-Gonzales et al. (2018) emphasize the importance of having concrete and measurable goals to make progress in combating CM. In the absence of national objectives for the prevention and follow-up of neglect, PHNs’ follow-up is at risk of becoming directionless, much like the findings in this study indicates.

## Strengths and Limitations of the Study

The trustworthiness was scrutinized in every phase of the research process in a continuous dialogue between a novice and a more seasoned qualitative researcher in a research community, in line with the hermeneutic approach and the chosen qualitative research design (Elo et al., 2014). We have attempted to clarify and make transparent all research phases, while continuously looking for alternative interpretations, ambiguities, and inconsistencies in a back-and-forth process (cf. Elo & Kyngäs, 2008; Elo et al., 2014). Presenting and discussing the preliminary findings during author dialogues and in research seminars and the final reporting have been based on trust and ethical primacy. A strength of the study is that the data is rich with practice narratives, not only general and abstract statements (Malterud, 2017). This made it possible to analyze both how the PHNs describe and explain their experiences and perceptions of the follow-up, and to create a view of their experiences of their practice based on their stories. We aimed for vividness in the presentation of faithful and rich descriptions that illuminate the categories (Elo et al. 2014).

It is possible that the PHNs who chose to participate in the FGs had a greater interest in, and knowledge about, neglect than the “average” PHN, which could potentially have influenced the findings (Elo et al. 2014). Alternative research designs and data collection, such as individual interviews, observations, and journals might have created even more nuanced findings. The use of vignettes can elicit more honest narratives (Hughes & Huby, 2001), and we therefore went rounds in advance to ensure that the vignette minimally introduced or reproduced our prior understanding. In line with Elo and Kyngäs’ (2008) content analysis process, we created a model based on the emerging categories, to help attain a condensed description of the phenomenon, and to serve future research.

## Implications

The findings of our study allow for professional self-reflection, supervision, and developing nursing care practices when there is concern about the child’s care at CHCs. Strengthening the PHNs’ knowledge of neglect is necessary, as PHNs experience that knowledge enhances their courage to act. The CHC must be organized so that responsibility for the vulnerable child is safeguarded through the prioritization of continuity. Appropriate resourcing of the CHC service and clearer guidelines are necessary for the PHNs to be able to follow-up the child’s care with more consistency and quality. PHNs should to a greater extent deliberately reflect on when child-centered care and the disease prevention perspective needs to be emphasized, as the findings show that the shift from family-centered care and the health promotion perspective can happen too late. Discussion in the public health nursing field should be encouraged, as well as a clarification with other institutions and professions, regarding the role PHNs should have in providing long-term follow-up when they encounter concern for care that is not subject to report to the CPS. It is important that neglect is explicitly addressed in national plans and in professional guidelines, rather than remaining hidden as a subtype of CM. The model can serve future research that should include the child perspectives, as well as parent- and sibling perspectives, in addition to placing a greater emphasis on social and economic factors and approaches at the population level. It is also important to study the public health nursing education program and CHC leadership.

## Conclusions

Findings show that public health nurses experience an internal negotiation process in the follow-up when they are concerned about the care for the child. Various prerequisites and approaches for follow-up including several types of dilemmas and prolonged dwellings characterized the follow-up. A lack of routines and goals, a dominant parental perspective, and an ambiguity around health promotion and disease prevention, all create challenges in PHNs’ follow-up. A model was created for further research in public health nursing to create knowledge that has the potential to prevent child neglect and promote child and family health.

## Supplemental Material

sj-docx-1-gqn-10.1177_23333936241267003 – Supplemental material for Public Health Nurses in an Internal Negotiation Process When There Is Concern About the Child’s Care Supplemental material, sj-docx-1-gqn-10.1177_23333936241267003 for Public Health Nurses in an Internal Negotiation Process When There Is Concern About the Child’s Care by Ingrid Elisabeth Mathisen Haaland and Terese Elisabet Bondas in Global Qualitative Nursing Research

sj-docx-2-gqn-10.1177_23333936241267003 – Supplemental material for Public Health Nurses in an Internal Negotiation Process When There Is Concern About the Child’s Care Supplemental material, sj-docx-2-gqn-10.1177_23333936241267003 for Public Health Nurses in an Internal Negotiation Process When There Is Concern About the Child’s Care by Ingrid Elisabeth Mathisen Haaland and Terese Elisabet Bondas in Global Qualitative Nursing Research

## References

[bibr1-23333936241267003] Albaek A. U. Kinn L. G. Milde A. M. (2018). Walking children through a minefield: How professionals experience exploring adverse childhood experiences. Qualitative Health Research, 28(2), 231–244. 10.1177/1049732317734828 29046119 PMC5734381

[bibr2-23333936241267003] Barrett E. O. Laholt H. Lorem G. F. Wang C. E. A. (2024). Exploring public health nurses’ acceptability of clinical assessment tools in a Norwegian child health centre. Primary Health Care Research & Development, 25, e10. 10.1017/S146342362400001X PMC10894717 38343358

[bibr3-23333936241267003] Bradbury-Jones C. Taylor J. Herber O. (2014). How theory is used and articulated in qualitative research: Development of a new typology. Social Science & Medicine, 120, 135–141. 10.1016/j.socscimed.2014.09.014 25241120

[bibr4-23333936241267003] Browne A. J. Doane G. H. Reimer J. MacLeod M. L. McLellan E . (2010). Public health nursing practice with “high priority” families: The significance of contextualizing “risk.” Nursing Inquiry, 17(1), 27–38. 10.1111/j.1440-1800.2009.00478.x 20137028

[bibr5-23333936241267003] Clément M.-E. Bérubé A. Chamberland C. (2016). Prevalence and risk factors of child neglect in the general population. Public Health, 138, 86–92. 10.1016/j.puhe.2016.03.018 27117500

[bibr6-23333936241267003] Coyne I. Holmström I. Söderbäck M. (2018). Centeredness in healthcare: A concept synthesis of family-centered care, person-centered care and child-centered care. Journal of Pediatric Nursing, 42, 45–56. 10.1016/j.pedn.2018.07.001 30219299

[bibr7-23333936241267003] Dahl B. M. (2020). Helsesykepleie: en grunnbok [Public health nursing: The basics]. Fagbokforlaget.

[bibr8-23333936241267003] Dahlbo M. Jakobsson L. Lundqvist P. (2017). Keeping the child in focus while supporting the family. Journal of Child Health Care, 21(1), 103–111. 10.1177/1367493516686200. 29156965

[bibr9-23333936241267003] Delmar C. (2021). Professionel sygeplejefaglig omsorg—en rammemodel. [Professional nursing care—A framework model]. Klinisk sygepleje, 35(3), 219–241. 10.18261/issn.1903-2285-2021-03-04

[bibr10-23333936241267003] Directorate of Health. (2024, April 28). Child health clinic and school health services. National Professional Guidelines. https://www.helsedirektoratet.no/retningslinjer/helsestasjons-og-skolehelsetjenesten/helsestasjon-05-ar

[bibr11-23333936241267003] Egeland B. (2009). Taking stock: Childhood emotional maltreatment and development psychopathology. Child Abuse & Neglect, 33, 22–26. 10.1016/j.chiabu.2008.12.004 19167068

[bibr12-23333936241267003] Einboden R. Rudge T. Varcoe C. (2019). Beyond and around mandatory reporting in nursing practice: Interrupting a series of deferrals. Nursing inquiry, 26(2), e12285. 10.1111/nin.12285 30801853

[bibr13-23333936241267003] Eklund A. L. Jangsten E. Gunnarsdóttir H. (2022). Assessing and promoting responsive interaction between parents and children – A qualitative study of the experiences of child health care nurses in Sweden. Journal of Pediatric Nursing, 63, e95–e101. 10.1016/j.pedn.2021.10.006 34688530

[bibr14-23333936241267003] Elo S. Kääriäinen M. Kanste O. Pölkki T. Utriainen K. Kyngäs H. (2014). Qualitative content analysis: A focus on trustworthiness. SAGE Open, 4(1), 1–10. 10.1177/2158244014522633

[bibr15-23333936241267003] Elo S. Kyngäs H. (2008). The qualitative content analysis process. Journal of Advanced Nursing, 62(1), 107–115. 10.1111/j.1365-2648.2007.04569.x 18352969

[bibr16-23333936241267003] Engström M. Hiltunen J. Wallby T. Lucas S. (2021). Child health nurses’ experiences of addressing psychosocial risk factors with the families they meet. Acta Paediatrica, 110(2), 574–583. 10.1111/apa.15492 32716528 PMC7891612

[bibr17-23333936241267003] Erisman J. C. de Sabbata K. Zuiderent-Jerak T. Syurina E. V. (2020). Navigating complexity of child abuse through intuition and evidence-based guidelines: A mix-methods study among child and youth healthcare practitioners. BMC Family Practice, 21, 1–11. 32738894 10.1186/s12875-020-01226-6 PMC7395977

[bibr18-23333936241267003] Felitti V. J. Anda R. F. Nordenberg D. Williamson D. F. Spitz A. M. Edwards V. Koss M. P. , et al. (1998). The relationship of adult health status to childhood abuse and household dysfunction. American Journal of Preventive Medicine, 14, 245–258. 9635069 10.1016/s0749-3797(98)00017-8

[bibr19-23333936241267003] Ferrara P. M. D. Corsello G. M. D. Basile M. C. M. D. Nigri L. M. D. Campanozzi A. M. D. Ehrich J. M. D. D. Pettoello-Mantovani M. M. D. P . (2015). The economic burden of child maltreatment in high income countries. The Journal of Pediatrics, 167(6), 1457–1459. 10.1016/j.jpeds.2015.09.044 26611458

[bibr20-23333936241267003] Gadamer H. G. (1989). Truth and method (2nd rev. ed.). Crossroad.

[bibr21-23333936241267003] Garner A. Yogman M. (2021). Preventing childhood toxic stress: Partnering with families and communities to promote relational health. Pediatrics, 148(2), e2021052582. https://doi.org/https://doi.org/10.1542/peds.2021-052582S 10.1542/peds.2021-052582 34312296

[bibr22-23333936241267003] Gilbert R. Widom C. S. Browne K. Fergusson D. Webb E. Janson S. (2009). Burden and consequences of child maltreatment in high-income countries. The Lancet, 373(9657), 68–81. 10.1016/S0140-6736(08)61706-7 19056114

[bibr23-23333936241267003] Hafstad G. S. Augusti E.-M. (2019). Ungdoms erfaringer med vold og overgrep i oppveksten. En nasjonal undersøkelse av ungdom i alderen 12 til 16 år [Young people’s experiences of violence and abuse in childhood. A national survey of young people aged 12 to 16 years]. Norwegian Centre for Traumatic Stress Studies. https://www.nkvts.no/content/uploads/2019/10/Rapport_4_19_UEVO.pdf

[bibr24-23333936241267003] Health Personnel Act. (1999). Act on Health Personnel, etc. (LOV-1999-07-02-64). Lovdata. https://lovdata.no/dokument/NL/lov/1999-07-02-64

[bibr25-23333936241267003] Health Research Act. (2008). Act on Medical and Health Research (LOV-2008-06-20-44). Lovdata. https://lovdata.no/dokument/NL/lov/2008-06-20-44

[bibr26-23333936241267003] Hildyard K. L. Wolfe D. A. (2002). Child neglect: Developmental issues and outcomes. Child Abuse & Neglect, 26(6–7), 679–695. 10.1016/S0145-2134(02)00341-1. 12201162

[bibr27-23333936241267003] Hughes R. Huby M. (2001). The application of vignettes in social and nursing research. Journal of Advanced Nursing, 37(4), 382–386. 10.1046/j.1365-2648.2002.02100.x. 11872108

[bibr28-23333936241267003] Kageyama M. Yokoyama K. (2018). Child-rearing support provided by public health nurses to people with mental illness: Qualitative descriptive study. The Open Nursing Journal, 12(1), 162–170. 10.2174/1874434601812010162 30197722 PMC6110077

[bibr29-23333936241267003] Kent S. Dowling M. Byrne G. (2011). Community nurses’ child protection role: Views of public health nurses in Ireland. Community Practitioner, 84(11), 33–36. 23270020

[bibr30-23333936241267003] Killén K. (2019). Barndommen varer i generasjoner: Forebygging er alles ansvar [Childhood lasts for generations: Prevention is everyone's responsibility] (4th ed.). KF.

[bibr31-23333936241267003] Liamputtong P. (2011). Focus group methodology: Principles and practice. SAGE.

[bibr32-23333936241267003] Lines L. E. Hutton A. Grant J. M. (2019). Navigating and negotiating meanings of child abuse and neglect: Sociocultural contexts shaping Australian nurses’ perceptions. Health & Social Care in the Community, 28(3), 941–949. 10.1111/hsc.12925 31833159

[bibr33-23333936241267003] Lines L. E. Kakyo T. A. Grant J. M. Hutton A. (2023). Invisibility of nurses and midwives in the public health response to child abuse and neglect: A policy review. Collegian, 30(2), 222–229. 10.1016/j.colegn.2022.09.002.

[bibr34-23333936241267003] Lines L. E. Kakyo T. A. Hutton A. E. Grant J. M. (2023). Nurses’ and midwives’ contributions to a public health response to keeping children safe from abuse and neglect: A Delphi study. Journal of Child Health Care. Advance online publication. 10.1177/13674935231153248 PMC11457461 36705058

[bibr35-23333936241267003] Lundén K. (2010). Att identifiera omsorgssvikt hos förskolebarn [To identify neglect in preschool children]. Stiftelsen Allmänna Barnhuset.

[bibr36-23333936241267003] Malterud K. (2017). Kvalitative forskningsmetoder for medisin og helsefag [Qualitative research methods for medicine and health sciences] (4th ed.). Universitetsforlaget.

[bibr37-23333936241267003] Martinsen K. (2003). Omsorg, sykepleie og medisin: historisk-filosofiske essays. [Care, nursing, and medicine: Historical-philosophical essays] (2nd ed.). Universitetsforlaget.

[bibr38-23333936241267003] Martinsen K. (2014). “Vil du meg noe?” Om sårbarhet og travelhet i helsevesenets rom [“Do you want something from me?” On vulnerability and busyness in the healthcare space]. In Alvsvåg H. Førland O. Jacobsen F. F. (Eds.), Rom for omsorg [Space for care] (pp. 225–245). Fagbokforlaget.

[bibr39-23333936241267003] McTavish J. R. Kimber M. Devries K. Colombini M. MacGregor J. C. Wathen C. N. MacMillan H. L. (2017). Mandated reporters’ experiences with reporting child maltreatment: a meta-synthesis of qualitative studies. BMJ Open, 7(10), e013942. 10.1136/bmjopen-2016-013942 PMC5652515 29042370

[bibr40-23333936241267003] Midtsund A. D. Garnweidner-Holme L. Valla L. Lukasse M. Henriksen L. (2023). A qualitative study of public health nurses’ experiences detecting and preventing child maltreatment in primary care settings. Journal of Advanced Nursing, 79(12), 4660–4671. 10.1111/jan.15761 37358075

[bibr41-23333936241267003] Mæland J. G. (2016). Forebyggende helsearbeid: folkehelsearbeid i teori og praksis [Preventive health care: Public health work in theory and practice] (4th ed.). Universitetsforlaget.

[bibr42-23333936241267003] Naughton A. M. Maguire S. A. Mann M. K. Lumb R. C. Tempest V. Gracias S. Kemp A. M. (2013). Emotional, behavioral, and developmental features indicative of neglect or emotional abuse in preschool children: A systematic review. JAMA Pediatrics, 167(8), 1–7. 10.1001/jamapediatrics.2013.192 23754198

[bibr43-23333936241267003] Neumann C. B. (2009). Det bekymrede blikket: En studie av helsesøstres handlingsbetingelser [The concerned gaze: A study of the conditions for action among public health nurses]. Novus.

[bibr44-23333936241267003] Nordanger D. Ø. Braarud H. C. (2014). Regulering som nøkkelbegrep og toleransevinduet som modell i en ny traumepsykologi [Regulation as the key concept and the window of tolerance as the model in a new trauma psychology]. Tidsskrift for Norsk psykologforening, 51(7), 530–536.

[bibr45-23333936241267003] Paavilainen E. Åstedt-Kurki P. (2003). Functioning of child maltreating families: Lack of resources for caring within the family. Scandinavian Journal of Caring Sciences, 17(2), 139–147. 10.1046/j.1471-6712.2003.00120.x 12753514

[bibr46-23333936241267003] Personal Data Act. (2018). Act on the processing of personal data (LOV-2018-06-15-38). Lovdata. https://lovdata.no/dokument/NL/lov/2018-06-15-38?q=personopplysning

[bibr47-23333936241267003] Poutiainen H. Hakulinen T. Laatikainen T. Kettunen T. (2015). Public health nurses’ concerns in preschool-aged children’s health check-ups. Journal of Research in Nursing, 20(7), 536–549. 10.1177/1744987115604660

[bibr48-23333936241267003] Ramiro-Gonzalez M. Dobermann D. Metilka D. Aldridge E. Yon Y. Sethi D. (2018). Child maltreatment prevention: A content analysis of European national policies. European Journal of Public Health, 29(1), 32–38. 10.1093/eurpub/cky176 PMC6345150 30184076

[bibr49-23333936241267003] Regulations on Child Health Clinics and School Health Services. (2018). Regulations on the municipality's health promotion and preventive work in child health clinics and school health services (FOR-2018-10-19-1584). https://lovdata.no/dokument/SF/forskrift/2018-10-19-1584.

[bibr50-23333936241267003] Research Ethics Act. (2017). Act on the organization of research ethics work (LOV-2017-04-28-23). Lovdata. https://lovdata.no/dokument/NL/lov/2017-04-28-23?q=forskningsetikk

[bibr51-23333936241267003] Rocha N. A. C. F. Silva F. P. S. dos Santos M. M. Dusing S. C. (2019). Impact of mother–infant interaction on development during the first year of life: A systematic review. Journal of Child Health Care, 24(3), 365–385. 10.1177/1367493519864742 31337225

[bibr52-23333936241267003] Schols M. W. A. de Ruiter C. Ory F. G. (2013). How do public child healthcare professionals and primary school teachers identify and handle child abuse cases? A qualitative study. BMC Public Health, 13(1), 1-16. 10.1186/1471-2458-13-807 24007516 PMC3847190

[bibr53-23333936241267003] Segal L. Armfield J. M. Gnanamanickam E. S. Preen D. B. Brown D. S. Doidge J. Nguyen H. (2021). Child maltreatment and mortality in young adults. Pediatrics, 147(1), e2020023416. 10.1542/peds.2020-023416. 33318224

[bibr54-23333936241267003] Shonkoff J. P. Garner A. S. (2012). The lifelong effects of early childhood adversity and toxic stress. Pediatrics, 129(1), E232–E246. 10.1542/peds.2011-2663 22201156

[bibr55-23333936241267003] SIKT. (2022, May 26). Notification form for personal data. Norwegian Agency for Shared Services in Education and Research. https://sikt.no/en/notification-form-personal-data

[bibr56-23333936241267003] Skarsaune K. Bondas T. (2016). Neglected nursing responsibility when suspecting child abuse. Clinical Nursing Studies, 4(1), 24–32. https://doi.org/https://doi.org/10.5430/cns.v4n1p24

[bibr57-23333936241267003] Söderman A. Jackson K. (2011). Barn som far illa i sin hemmiljö—BVC-sjuksköterskors upplevelser av att möta och hjälpa barnen [Children who are harmed in their home environment—Public health nurses’ experiences of encountering and helping the children]. Nordic Journal of Nursing Research, 31(4), 38–42. 10.1177/010740831103100408

[bibr58-23333936241267003] Sroufe L. A. Egeland B. Carlson E. A. Collins W. A. (2005). The development of the person: The Minnesota study of risk and adaption from birth to adulthood. Guilford Press.

[bibr59-23333936241267003] St. Marie B. Jimmerson A. Perkhounkova Y. Herr K . (2021). Developing and establishing content validity of vignettes for health care education and research. Western Journal on Nursing Research, 43(7), 677–685. 10.1177/0193945920969693 PMC8096852 33150841

[bibr60-23333936241267003] Stoltenborgh M. Bakermans-Kranenburg M. J. van IJzendoorn M. H. (2013). The neglect of child neglect: a meta-analytic review of the prevalence of neglect. Social Psychiatry and Psychiatric Epidemiology, 48(3), 345–355. 10.1007/s00127-012-0549-y 22797133 PMC3568479

[bibr61-23333936241267003] Thoresen S. Myhre M. Wentzel-Larsen T. Aakvaag H. F. Hjemdal O. K. (2015). Violence against children, later victimisation, and mental health: A cross-sectional study of the general Norwegian population. European Journal of Psychotraumatology, 6(1), 26259. 25591729 10.3402/ejpt.v6.26259 PMC4296052

[bibr62-23333936241267003] UNICEF. (1989). Convention on the rights of the child. https://www.unicef.org/child-rights-convention/convention-text

[bibr63-23333936241267003] Visscher S. J. A. van Stel H. F. (2017). Variation in prevention of child maltreatment by Dutch child healthcare professionals. Child Abuse & Neglect, 70, 264–273. 10.1016/j.chiabu.2017.05.020 28641135

[bibr64-23333936241267003] WHO. (2022, September 19). Child maltreatment. World Health Organization. https://www.who.int/news-room/fact-sheets/detail/child-maltreatment

[bibr65-23333936241267003] Wightman L. Hutton A. Grant J. (2022). Child and family health nurses' roles in the care of infants and children: A scoping review. Journal of Child Health Care, 26(3), 448–460. 10.1177/13674935211026123 34116592

[bibr66-23333936241267003] World Medical Association. (2013). WMA Declaration of Helsinki—Ethical principles for medical research involving human subjects. World Medical Association. https://www.wma.net/policies-post/wma-declaration-of-helsinki-ethical-principles-for-medical-research-involving-human 10.1001/jama.2013.281053 24141714

