# Supplementary material for: Public Health Nurses in an Internal Negotiation Process When There Is Concern About the Child’s Care
Source: Glob Qual Nurs Res. 2024 Aug 24;11:23333936241267003. doi: 10.1177/23333936241267003 (PMC11344900; doi:10.1177/23333936241267003)
Supplement: sj-docx-1-gqn-10.1177_23333936241267003 – Supplemental material for Public Health Nurses in an Internal Negotiation Process When There Is Concern About the Child’s Care [file sj-docx-1-gqn-10.1177_23333936241267003.docx]

Supplementary file 1. The interview guide

**Public health nurses’ follow-up of children and families when there is concern about the child’s care (Ingrid Mathisen Haaland & Terese Bondas)**

Introduction: I would like to hear how you follow up with the child and the parents over time when there are concerns about the care the child is receiving. The legislation states that healthcare professionals have a duty to report to the child welfare services when *serious* neglect is suspected. I want to know how you follow up when you encounter less severe neglect that is not covered by mandatory reporting.

Vignette: I will get you started with this vignette. What thoughts or associations do you get?

Interview questions:

1. Can you tell me about a specific situation where you have experienced concern for the care?
   - What did you see/sense/observe?
2. Can you tell me about your experiences with different approaches when you have been concerned that a child is not receiving adequate care?
   - What approaches and attitudes would you highlight as useful/important in your interactions with families?
3. When you are concerned about the care, how do you follow up with the family?
   - What specifically do you do?
   - How do you follow up with the child?
   - How do you follow up with the parents?
   - Do you address the concerns with the parents? How do you address the concerns with the parents?
   - How do you document when there are concerns about the care?
   - How is the follow-up for those who do not attend?
   - How do you experience the relationship and collaboration with the parents?
4. How do you experience the care provided to children living in neglectful environments at the child health clinic? (Is the CHC a relevant place to help them?)
5. How do you experience that personal experiences play into the individuals’ way of following up?
6. What professional knowledge do you find useful/relevant in following up and understanding neglect?

….Can you tell more about that? ….Concrete example? …What experiences do you have with what X is talking about?
