# Supplementary material for: Public Health Nurses in an Internal Negotiation Process When There Is Concern About the Child’s Care
Source: Glob Qual Nurs Res. 2024 Aug 24;11:23333936241267003. doi: 10.1177/23333936241267003 (PMC11344900; doi:10.1177/23333936241267003)
Supplement: sj-docx-2-gqn-10.1177_23333936241267003 – Supplemental material for Public Health Nurses in an Internal Negotiation Process When There Is Concern About the Child’s Care [file sj-docx-2-gqn-10.1177_23333936241267003.docx]

**Supplementary file 2.**

Overview of categories: Public health nurses follow-up when concerned for the child’s care - An internal negotiation process

|  |  |  |  |
| --- | --- | --- | --- |
| Public health nurses’ follow-up when concerned for care:  An internal negotiation process | | | |
| Prerequisites | Approaches | Dilemmas | Dwellings |
| Time  Continuity  Scientific knowledge  Professional and personal experience  Personal characteristics Documentation | Observing, interpreting, and assessing  Acting  Closer follow-up  Communicating concerns with the parents  Guidance  Cooperation  Follow-up on non-attendance | Negotiating perspectives  Doubt  Building relationships vs. child advocacy  Health-promoting role vs. protecting children  Trust vs. distrust  Reporting to CWS | Scrutinizing myself  Outcomes |
